# Alveolar ridge preservation in defect sockets in the maxillary aesthetic zone followed by single-tooth bone level tapered implants with immediate provisionalization: a 1-year prospective case series

**DOI:** 10.1186/s40729-021-00292-4

**Published:** 2021-02-19

**Authors:** Caroliene M. Meijndert, Gerry M. Raghoebar, Arjan Vissink, Henny J. A. Meijer

**Affiliations:** 1 Department of Oral and Maxillofacial Surgery, University Medical Center Groningen, University of Groningen, PO Box 30.001, NL-9700 RB Groningen, The Netherlands; 2 Department of Implant Dentistry, Dental School, University Medical Center Groningen, University of Groningen, Groningen, The Netherlands

**Keywords:** Aesthetic region, Socket preservation, Implant design, Bone loss

## Abstract

**Background:**

Clinical studies of single-tooth replacement in compromised bone using bone level tapered implants in the aesthetic zone are scarce.

**Aim:**

To assess clinically, radiographically and aesthetically over 1 year the performance of a bone level tapered implant in the maxillary aesthetic zone in sites after alveolar ridge preservation.

**Material and methods:**

Thirty patients (16 male, 14 female) with a failing tooth and large bone defect after removal received alveolar ridge preservation. After 3 months, implants were placed with immediate provisionalization. Definitive restorations were placed after 3 months. The treatment was evaluated 1 year following the definitive restoration.

**Results:**

All the patients attended the 1-year follow-up. One implant was lost (96.7% implant survival rate). The mean implant stability quotient value was 68.9 ± 8.74 at implant placement. The mean marginal bone level change was minor (− 0.07 ± 0.12 mm). The mean mid-buccal mucosa changed with + 0.01 ± 0.45 mm. The median Pink Esthetic Score and White Esthetic Score after 1 year were 6 [4; 7] and 8 [7; 9], respectively. The patients’ mean overall satisfaction (0–100 VAS scale) was 86.6 ± 10.3.

**Conclusion:**

Bone level tapered implants with immediate provisionalization perform well after alveolar ridge preservation in the maxillary aesthetic zone, according to implant stability, clinical, radiographic, aesthetic and patient-centred outcomes.

**Trial registration:**

NTR, NL8755. Registered on 1 January 2016

## Introduction

Immediate implant placement is frequently advocated for a single failing tooth in the maxillary aesthetic zone. However, in some cases, after tooth extraction, the future implant site is inadequate for primary implant placement [1, 2]. Examples of inadequate sites for immediate implant placement are large bone deficiencies, severe recession of the mucosa and extensive infection [3]. Just removing the tooth and letting the alveolus heal without extra precautions can often lead to progressive physiologic resorption of the buccal bony wall [4]. A few techniques have been described to avert this ‘physiologic collapse’ of the alveolar ridge after tooth extraction, including alveolar ridge preservation [3]. Alveolar ridge preservation cannot stop the resorption process, but it can reduce the degree of alveolar alteration [5, 6]. This is particularly important in the aesthetic region where the alveolar bone supports the mucosa, which determines a great deal of the aesthetic outcome of implant restoration. The Jung et al. [3] systematic review noted that alveolar ridge preservation has clear advantages in preserving alveolar ridge volume in the aesthetic region.

There is a wide range of dental implant designs, one of which is a tapered implant body. In 2018, Jokstad and Ganeles defined a tapered implant as a *cylindrical implant where the endosseous part narrows in diameter towards the apex* [7]. A possible benefit of a tapered implant design is improved primary stability compared to parallel-walled implants [8, 9]. Improved primary stability is particularly important in low-density and soft bone and in healing extraction sockets. Furthermore, it is said that there is less risk of bone fenestrations in anatomical undercuts at the apical part of tapered implants, as often is present with the maxillary alveolar processes [9]. Good results have been achieved with tapered implants in the aesthetic region including implant survival, bone level change and aesthetic outcome, both in preserved and in non-preserved sites [10–13]. Various tapered implant brands are available, but in 2015, a new line was launched: the Straumann Bone Level Tapered Implant system (BLT). It has a tapered body, converging from the cervical part to the apex, and is further equipped with the established characteristics of previous Straumann designs such as a SLActive surface [14] and platform switch with conical implant/abutment connection [15]. To the best of our knowledge, only a few studies have reported the use of this newly designed implant system in compromised bone, and rarely with measurement of primary implant stability [16–18]. A full-scale assessment (primary stability, marginal bone level change, change in clinical peri-implant parameters, aesthetic outcome, patient-reported outcomes) on the performance of the Straumann BLT implant system with immediate provisionalization in the maxillary aesthetic area has not yet been reported. Therefore, the purpose of this prospective observational case series was to perform a full-scale assessment of the clinical, radiographic, aesthetic and patient-reported outcomes of single-tooth bone level tapered implants with immediate provisionalization after alveolar ridge preservation in the maxillary aesthetic zone over a 1-year follow-up period.

## Material and methods

### Study design

The study was designed as a prospective clinical case series on 30 consecutive patients requesting implant treatment for a single failing tooth in the maxillary aesthetic zone but for whom immediate implant placement was not possible. Recruitment and inclusion of patients took place at the Department of Oral and Maxillofacial Surgery of the University Medical Center Groningen, The Netherlands, from January 2016 to December 2017. The research protocol was approved by the Medical Ethics committee of the UMCG (METc 2015.517). The trial was registered in the Dutch Trial Register (trial ID: NL8755). Informed consent was signed by all the participants prior to treatment. This manuscript was constructed according to the STROBE guidelines for cohort studies [19].

### Participants

The inclusion criteria were as follows:

- Treatment site in the anterior maxilla (P1to P1).
- A single failing tooth and in need of ridge preservation prior to implant placement because of a vertical buccal bone wall defect of > 5 mm of the extraction socket, assessed after tooth extraction by bone sounding technique.
- Presence of natural teeth on both sides of the planned implant site.
- At least 18 years of age at the time of treatment.
- The patient is capable of understanding and giving informed consent.

Patients were excluded when there was:

- Presence of active clinical periodontal disease as expressed by probing pocket depths ≥ 4 mm combined with bleeding on probing in the natural dentition other than the affected tooth
- Smoking
- A history of radiotherapy to the head and neck region
- Use of bisphosphonates less than 10 years ago

### Treatment procedures

Participants with a failing tooth and not suitable for immediate implant placement were eligible for this study. They were informed about and consented to the research protocol prior to tooth extraction. All the participants started antibiotic therapy the day before the extraction (amoxicillin 500 mg, 3 times daily for 7 days, or clindamycin 300 mg, 4 times daily for 7 days in case of an amoxicillin allergy) and a 0.2% chlorhexidine mouth rinse, twice daily for 7 days. After administering local anaesthesia, the tooth was removed as atraumatically as possible using a periotome and forceps. The socket was cleaned, and the granulation tissue was removed. A bone graft, harvested from the maxillary tuberosity or retromolar area, was shaped to fit the labial bone wall defect and inserted in the alveolus. Next, the socket was augmented with a mixture of autologous bone and anorganic bovine bone (Geistlich Bio-Oss®, Geistlich Pharma AG) [20]. The alveolus was closed with a mucosa graft from the maxillary tuberosity or palate. Wound healing and suture removal were checked 2 weeks after the surgery. The participants wore a removable partial denture during the healing period. The implants were placed 3 months after the augmentation procedure.

One day before implant placement, all the participants started antibiotic therapy (amoxicillin 500 mg, 3 times daily for 7 days, or clindamycin 300 mg, 4 times daily for 7 days in case of an amoxicillin allergy) and a 0.2% chlorhexidine mouth rinse, twice daily for 7 days. All the surgeries were performed by the same, experienced, surgeon (GMR). After administering local anaesthesia, a mid-crestal incision with a divergent reliving incision was made next to the distal tooth for a small muco-periosteal flap elevation. The implant position was dictated by a (semi-guided) surgical template, and the implant bed was prepared following the manufacturers’ surgical guidelines. All the participants received a Straumann Bone Level Tapered (BLT) implant (Institute Straumann AG, Basel, Switzerland) according to the pre-operative planning. The implants were installed 3 mm apically of the prospective gingival margin of the future restoration, with an insertion torque of at least 45 Ncm. If the thickness of the labial bone wall was < 2 mm (Grunder et al. [21]), a 1:1 mixture of autologous bone (from the drills) and bovine bone (Cerabone®, Botiss Biomaterials GmbH, Zossen, Germany) was augmented. The augmented area was covered with a collagen membrane (Collprotect®, Botiss Biomaterials GmbH, Zossen, Germany).

Following implant placement, an implant-level open tray impression was made with a vinylpolysiloxane precision impression material (Provil Novo, Medium Fast Set, Kulzer Mitsui Chemical Group, Germany) (HJAM). The impression was sent to the dental laboratory which then manufactured a screw-retained provisional restoration. The provisional restoration consisted of a platform-switched titanium stock temporary abutment with an acrylic resin restoration. The participants received the provisional restoration on the day the implant was placed. The screw of the provisional restoration was torqued to 25 Ncm. After 3 months, an impression was made for the construction of a definitive crown. Definitive porcelain fused to zirconia restorations was cemented onto individualized zirconium abutments with a platform-switched, internal conical connection (zirconium CARES® abutment) or designed as screw-retained restorations with a titanium base (Variobase® for single crowns AS, Straumann AG, Basel). In both cases, the implant screws were tightened with a torque of 35 Ncm. Oral hygiene instructions were given after installing both the provisional and the definitive restorations.

### Evaluation

Clinical, radiographic, photographic and patient-centred outcomes were assessed before implant placement (*T*_pre_), 1 month after definitive restoration placement (*T*_1_) and 12 months after definitive crown placement (*T*_12_). All the assessments were done by the same observer (CMM).

#### Outcome measures

- At the removal of the failing tooth, the size of the labial bone wall defect was measured, and the origin of the donor bone (donor site), used to preserve the alveolar ridge, was noted.
- At implant placement, it was noted if the thickness of the labial bone wall was insufficient, being < 2 mm [21], and additional augmentation was needed.
- Implant stability was analysed by measuring the initial fixation (Implant Stability Quotient (ISQ)) using an Osstell™ mentor device (IntegrationDiagnostics AB, Gothenburg, Sweden) [22, 23]. Implant stability was measured immediately after implantation and just before placement of the definitive restoration.
- Implant survival was defined as the percentage of the implants that are in place and functional at the time of follow-up.
- Marginal bone level change: the bone level was measured by a trained observer (CMM) on standard peri-apical radiographs with individually fitting aiming devices [24]. The distance from the implant shoulder to the first bone to implant contact was taken on the mesial and distal side of the implant. Only the bone level changes apical from the implant shoulder were taken into account. Any bone that was depicted above the implant shoulder was given a value of 0.00 mm.
- Probing depth: the implant and the adjacent teeth were probed at four sites (mesial, distal, buccal, palatal). The probing depth was measured to the nearest 1 mm using a periodontal Click-probe® with a standard pressure of 0.2–0.25 N (KerrHawe Dental Corporation, Bioggio, Switzerland).
- Modified plaque index [25], modified sulcus bleeding index [25], gingiva index [26].
- Aesthetic parameters according to the modified Pink and White Esthetic Score (PES-WES) [27].
- The mid-buccal mucosa and papilla change [10] were measured on intra-oral colour photographs using a periodontal probe parallel to the axis of the implant crown for calibration (Williams Color-Coded probe; Hu-Friedy Chicago, IL, USA). A horizontal line was drawn between the incisal line of the adjacent teeth (reference line). The distance between the reference line and the mesial and distal papilla and the mid-buccal mucosal margin was measured and analysed (Adobe Photoshop 21.0.3 2020, Adobe Systems Inc., San Jose, USA).
- Participant satisfaction was assessed with a questionnaire used by Den Hartog et al. [11] prior to implant placement and 1 year after definitive crown placement. The questions focussed on the influence of the affected site or the new implant restoration on the patient self-confidence, function and aesthetics, and had to be answered on a 5-point scale, ranging from very dissatisfied (score 1) to very satisfied (score 5). The overall satisfaction was measured on a 0–100 visual analogue scale (VAS).
- Complications: any biological and technical complications that occurred from surgery up to 1 year after definitive crown placement were recorded.

### Statistical analysis

All the data was checked for normality using QQ plots and the Shapiro-Wilk test in order to determine the appropriate statistical method.

Ordinal and not-normally distributed continuous data was analysed using the Wilcoxon signed-rank test to determine any significance between time intervals. The normally distributed data was analysed using the paired *t* test to determine any significance between time intervals. McNemar’s test was used for the questions in the questionnaire. A *p* value of 0.05 was considered to indicate statistical significance. All the analyses were performed using SPSS (PASW Statistics 23.0, SPSS Inc.; IBM Corporation, Chicago, IL, USA).

## Results

A total of 30 participants were included. The characteristics of the study population are depicted in Table 1. No patients dropped out or were lost to follow-up.

**Table 1:** Baseline characteristics

Number of participants	30
Age (years), mean ± SD (range)	43 ± 16 (18–74)
Male/female ratio	16/14
Labial bone wall defect (mm), mean (range)	8.6 (5–12)
Implant location (I_1_/I_2_/C/P_1_)	24/5/1/0
Implant length (mm) (10/12/14)	0/19/11
Implant diameter (mm) (3.3/4.1)	4/26

### Labial bone wall defect

In all cases, the defect of the labial bone wall after removing the tooth was U-shaped. Five participants had also a fistula at the labial mucosa. The origin of the bone (donor site) to preserve the alveolar ridge was the maxillary tuberosity in 14 cases and the retromolar area in 16 cases. The choice for the retromolar area was made in case wisdom teeth were present in the maxilla. When maxillary wisdom teeth are present, usually, less bone can be harvested in these areas.

### Insufficient labial bone wall thickness at implant placement

In all cases, there was enough bone volume after alveolar ridge preservation to insert the implant with sufficient primary stability. The thickness of the labial bone wall at implant placement was < 2 mm in 10 cases. In 5 of these cases, the alveolar ridge preservation was previously done with bone from the maxillary tuberosity, whereas in the other 5 cases, the bone was harvested from the retromolar area.

### Implant stability

The mean implant stability immediately after implantation was 68.9 ± 8.74 ISQ and 80.2 ± 2.73 ISQ just before placement of the definitive restoration.

### Survival rate

One patient lost the implant 2 months after placement. This resulted in an implant survival rate of 96.7%. The patient was excluded from further analysis (Table 2).

**Table 2:** Survival rate (%) and bone level change from 1 month (*T*_1_) to 12 months (*T*_12_) after definitive restoration placement measured in millimetres, with the median [IQR] and mean ± SD depicted in the parentheses

	*n* = 29
Implant loss (survival rate %)	1 (96.7%)
Bone level change mesial	0.00 [− 0.04; 0.00] (− 0.07 ± 0.16)
Bone level change distal	0.00 [− 0.02; 0.00] (− 0.06 ± 0.14)
Bone level change mesial + distal	0.00 [− 0.08; 0.00] (− 0.07 ± 0.12)

### Peri-implant bone level change

**Fig. 1:**
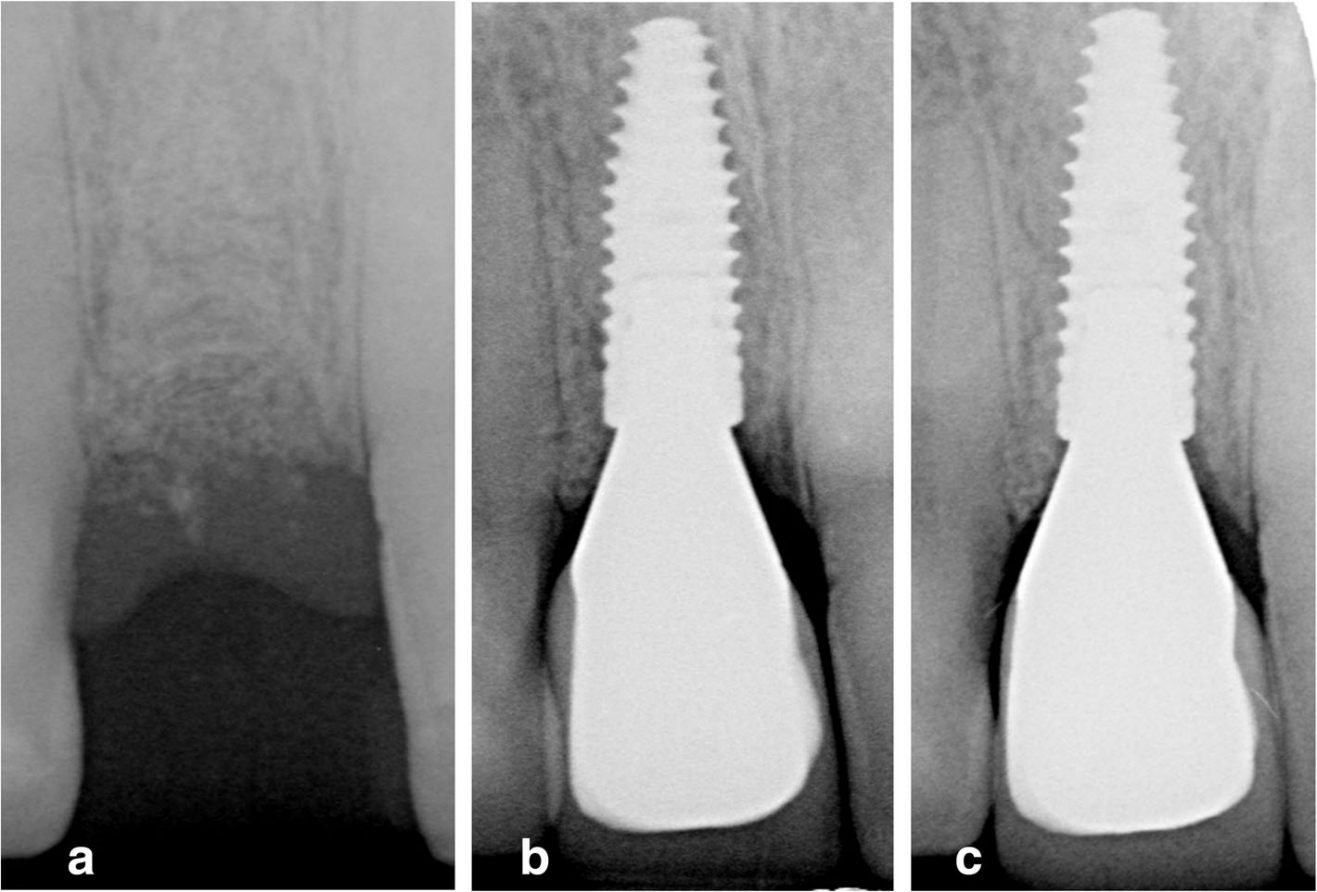
Bone level. **a** Prior to implant placement (3 months after preservation). **b** One month after definitive restoration placement. **c** Twelve months after definitive restoration placement

The main results are depicted in Table 2. There were statistically no significant changes from *T*_1_ to *T*_12_ (Fig. 1). The peri-implant bone level changes were not normally distributed, so only the medians and IQR were calculated. To give more insight into the actual change, bone level changes are also depicted in mean ± SD. The mean bone level change was − 0.07 ± 0.16 mm on the mesial side and − 0.06 ± 0.14 on the distal side.

### Clinical parameters

The bleeding index, gingiva index and probing depth scores are depicted in Table 3. There were no significant differences in the parameters between the *T*_1_ and *T*_12_ follow-up periods.

**Table 3:** Clinical parameters at 1 month (*T*_1_) and 12 months (*T*_12_) after definitive crown placement (*n* = 29)

	*T*_1_	*T*_12_	Significance
Pocket probing depth in millimetres, mean ± SD			
Mesial side	3.7 ± 1.7	3.9 ± 1.7	*p* = 0.56
Mid-buccal side	3.2 ± 1.0	3.0 ± 1.0	*p* = 0.26
Distal side	4.1 ± 1.6	4.3 ± 1.7	*p* = 0.42
Palatal side	3.4 ± 1.2	3.2 ± 1.2	*p* = 0.29
Mean of all sides	3.4 ± 1.1	3.6 ± 1.1	*p* = 0.35
Bleeding index, median [IQR]	1 [0; 2]	0 [0; 2]	*p* = 0.25
Gingiva index, median [IQR]	0 [0; 0]	0 [0; 0]	*p* = 0.56

### Aesthetic evaluation

**Fig. 2:**
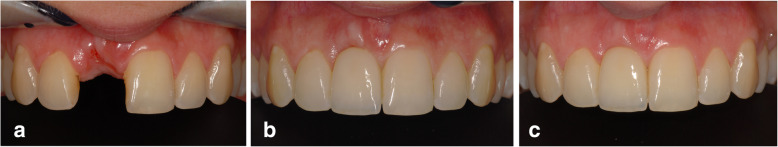
Soft tissue changes. a Pre-implant situation. **b** One month after definitive restoration placement. **c** Twelve months after definitive restoration placement

Aesthetics were evaluated using the modified PES/WES index and mucosa level (Fig. 2). A summary of the outcomes is depicted in Table 4. At *T*_12_, the median PES was 6 [4; 7], and median WES was 8 [7; 9]. There were no significant differences between the *T*_1_ and *T*_12_ follow-up periods. The mid-buccal mucosa remained stable between the 1st month and the 12th month after definitive restoration placement (+ 0.01 ± 0.45, *p* = 0.91).

**Table 4:** Evaluation of the aesthetic outcome at *T*_12_ (PES/WES) and mucosa level change from *T*_1_ to *T*_12_ (*n* = 29)

Aesthetic evaluation at *T*_12_	
PES	6 [4; 7]
WES	8 [7; 9]
Mucosa level changes from *T*_1_ to *T*_12_ in millimetres, mean ± SD	
Mid-buccal mucosa	+ 0.01 ± 0.45
Mesial papilla	+ 0.13 ± 0.55
Distal papilla	+ 0.17 ± 0.54

### Patient satisfaction

At baseline, before surgery, the patients gave an overall satisfaction score of 54.9 ± 21.0 on the VAS scale (Table 5). This score increased significantly after the definitive implant restoration was placed, up to a mean score of 86.6 ± 10.3 at the *T*_12_ follow-up (*p* < 0.001). Items of function and presence of shame improved significantly between *T*_pre_ and *T*_12_.

**Table 5:** Patient satisfaction was rated on a 0–100 VAS scale and further clarified through a questionnaire. *T*_pre_ is the time prior to implant placement. *T*_12_ is 12 months after definitive crown placement

	*T*_pre_, *n* = 28^a^	*T*_12_, *n* = 29	*p* value
**Overall patient satisfaction**, mean ± SD			
**VAS scale**	54.9 ± 21.0	86.6 ± 10.3	< 0.001^¥^
**Questionnaire**, % in agreement			
**Well-being**			
Presence of shame	21.4	0	0.03^‡^
Decreased self-confidence	7.1	0	0.50^‡^
Aware of prosth. visibility	21.4	6.9	0.29^‡^
**Function**			
Evade eating with affected zone	67.9	32.1	0.02^‡^
Decreased chewing ability	57.1	6.9	0.00^‡^
Influences speech	60.7	0	0.00^‡^
Influences taste	64.3	0	0.00^‡^
**Aesthetics**			
Satisfied with the shape of the RPD/crown	85.7	75.9	0.75^‡^
Satisfied with the colour of the RPD/crown	96.4	96.6	1.00^‡^
Satisfied with the shape of the mucosa	92.3	100	0.50^‡^
Satisfied with the colour of the mucosa	88.5	96.6	0.63^‡^

^a^One implant was lost between *T*_pre_ and *T*_12_. One patient did not wear the partial denture and has therefore not answered the questionnaire. This individual was left out of the analysis

^¥^Paired *t* test

^‡^McNemar’s test

### Complications

One patient lost the implant during the osseointegration period. A specific reason for this could not be found. This patient was successfully treated again after a healing period of 3 months. Other complications were minor: four participants reported they had lost some bone granules from the applied augmentation at the routine check-up 2 weeks after surgery (no further treatment was necessary), and 2 participants reported mobility of the provisional restoration 2 weeks after placement (the screws could be retightened again without further complications). There were no complications after the definitive restorations were placed.

## Discussion

This 1-year prospective case series reports the results of solitary Straumann Bone Level Tapered (BLT) implants in the aesthetic zone placed after alveolar ridge preservation and in combination with immediate provisionalization. Apart from one implant being lost in the early stages of osseointegration, there was a good primary stability at implant placement of all implants, marginal bone loss was minor, peri-implant soft-tissues were stable and patients and professionals were satisfied with the aesthetic results.

To the best of our knowledge, only the Caiazzo et al. [16] pilot study has published information on the Straumann BLT implant in the aesthetic region. However, they only reported the changes in buccal bone thickness and had a 6-month follow-up. Two other studies, though, focussed on Straumann BLT implants in the molar regions. Levine et al. [17] noted a survival rate of 98.3% and a marginal bone level change of − 0.3 ± 0.46 mm after 1 year, which is slightly higher than that described by us (− 0.07 ± 0.12 mm). However, baseline measurements were made immediately after implant placement, in contrast to our baseline measurement, which was 4 months after implant placement. Pariente et al. [18] described that most bone loss occurs in the 3 months after implant surgery. Here, the mean bone loss during the first year after implant placement was 0.35 ± 0.23 mm, of which 0.28 ± 0.19 mm in the first 3 months. This could explain the difference in the results between our and the above-mentioned studies.

The implant stability quotient was measured in neither the study of Levine et al. [17] nor the study of Pariente et al. [18]. Moroi et al. [28] studied implant stability of tapered implants versus cylindrical implants, but these implants were from a different brand and mostly placed in the posterior region. They found a mean ISQ value of 60.2 ± 12.41 at implant placement and 66.6 ± 9.00 at definitive crown placement for the tapered implants. These values were higher than for the cylindrical implants, being 54.7 ± 7.92 and 64.0 ± 5.78 ISQ at respectively implant placement and definitive crown placement. The rationale to use tapered implants is the claimed less risk of bone fenestrations in anatomical undercuts at the apical part of tapered implants, as often it is present with the maxillary alveolar processes. In the literature, it is claimed that a tapered implant design has the possible benefit of improved primary stability compared to parallel-walled implants. However, the question was if this is also true in the relatively soft bone as is the case in alveolar ridge preservation sites, because initial implant stability measurements in the maxillary aesthetic region were not performed. This is the reason why implant stability has been added as an outcome measure in the present study. In the present study, the mean ISQ values were high, being respectively 68.9 ± 8.74 and 80.2 ± 2.73, indicating good implant stability at both evaluation time points for this new implant design in the maxillary aesthetic region in sites in which alveolar ridge preservation has been performed.

In the literature, waiting times to place implants after alveolar ridge preservation differ from 3 to 6 months [29]. A long waiting time gives the risk of significant ridge alterations and resorption of the applied augmentation [1, 5]. A short waiting time gives the risk of placing the implant in soft bone, thus not reaching enough initial implant stability. In the present study, it was chosen to have a healing time of 3 months after alveolar ridge surgery, following the protocol of previous studies on alveolar ridge preservation at the same department in which good results were reached [12, 30].

The thickness of the labial bone wall at implant placement was insufficient in 10 cases (33.3%) and had to be augmented to achieve a labial bone wall thickness of at least 2 mm. The insufficient thickness could not be due to the type of bone used for the alveolar ridge preservation because the origin of augmented bone was evenly distributed between maxillary tuberosity and retromolar area. Apparently, placement of tapered implants in sites in which alveolar ridge preservation has been executed does not prevent the need for an extra bone augmentation at the labial side of the implant in all cases. This was also seen in other studies with alveolar ridge preservation in the aesthetic region. In Zuiderveld et al. [30], in 45% of the cases, an extra bone augmentation procedure was needed, and in Lai et al. [31], this was the case in 26.3%.

In the absence of studies with the same implant brand and design, comparisons can best be made with other implant systems with a tapered design, applied in the aesthetic region. Eghbali et al. [13] presented 1-year results of alveolar ridge preservation and connective tissue grafts. In this study, the implants (NobelActive implant system, Nobel Biocare AB, Goteborg, Sweden) were placed 4 months after alveolar ridge preservation, and a provisional restoration combined with connective tissue grafts was performed 3 months later. Implant survival after 1 year was 100%. Favourable clinical and aesthetic outcomes were reported. The mean marginal bone loss was 0.53 mm, and the mid-facial recession amounted 0.05 mm at 1 year. In the Zuiderveld et al. [30] study, the NobelReplace CC implant system (Nobel Biocare AB, Goteborg, Sweden) was used in the maxillary aesthetic region, again after ridge preservation. After a 1-year follow-up, the implant survival was 100%, and the change in marginal bone level was + 0.03 ± 0.4 mm mesially and + 0.13 ± 0.5 mm distally. These changes are minor and are comparable to those in the present study.

The aesthetic outcome is of particular interest for restorations in the aesthetic zone. Aesthetics can be evaluated by professionals with the PES/WES score [27] and by patients with questionnaires [32]. Our median PES/WES score was 6/8 after 1 year. This is in line with Zuiderveld et al. [30] who noted a mean PES/WES score of 6.6/8.7 after 1 year. The present study’s overall patient’s satisfaction score was also very much alike with the satisfaction scores mentioned by Zuiderveld et al. [30]. It is remarkable that PES was rated lower than the WES by the professional although the mucosa was better valued than the crown by the patients. This is probably due to the fact that professionals rate other items as important than patients. In both our and Zuiderveld’s study [30], patients are more satisfied with the end result than the professional observers. This phenomenon has been recorded before, showing little correlation between the professional’s opinion and the patient’s opinion about the aesthetics [33]. Hence, it is not justified to leave out one of them in evaluating the aesthetic outcome. Nonetheless, the overall satisfaction with the implant restorations was high. This is in accordance with other reports on single implants in the aesthetic zone [10, 12].

The question is if peri-implant bone level and mid-buccal mucosa level will remain stable in sites in which alveolar ridge preservation has been performed. The aforementioned study by Eghbali et al. [13] also presented 5-year results and reported a peri-implant bone loss of 0.47 mm which is remarkably stable compared to 1 year. Next to this, only a small extra recession, being 0.07 mm, of the mid-buccal mucosa level took place. If the implant system in the present study acts the same as the one used in the study of Eghbali et al. [13], the prognosis is good towards 5-year results. However, despite both implant systems are tapered, one cannot automatically assume that also results will be the same. Both implant systems have their specific characteristics and surface topography. Therefore, it is worthwhile to extend the follow-up time of the present study to at least 5 years.

## Conclusion

Within the limitations of this case series, it can be concluded that the Straumann Bone Level Tapered implant system, applied in the maxillary aesthetic region after alveolar ridge preservation and in combination with immediate provisionalization, is accompanied with a high survival rate, stable marginal bone levels and soft tissue levels, good aesthetic outcomes and high patient satisfaction 1 year after implant placement.

## Data Availability

The datasets used and/or analysed during the current study are available from the corresponding author on reasonable request.
